# Endovascular treatment and clinical outcomes in minor ischemic stroke due to large-vessel disease: a retrospective study

**DOI:** 10.3389/fnins.2026.1885422

**Published:** 2026-08-21

**Authors:** Xiang-Jie Gao, Li-Hua Ren

**Affiliations:** 1 Department of Neurology, The Affiliated Yongchuan Hospital of Chongqing Medical University, General Practice School of Chongqing Medical University, Chongqing Key Laboratory of Cerebrovascular Disease Research, Chongqing, China; 2 Department of Intensive Care Unit, The Affiliated Yongchuan Hospital of Chongqing Medical University, General Practice School of Chongqing Medical University, Chongqing, China

**Keywords:** collateral circulation, early neurological deterioration, endovascular treatment, intracranial stenosis, ischemic penumbra, large-vessel occlusion, minor ischemic stroke

## Abstract

**Background:**

Patients with minor ischemic stroke may still experience early neurological deterioration when severe intracranial stenosis or occlusion is present. The optimal role of endovascular treatment (EVT) in this imaging-defined population remains uncertain. This study aimed to evaluate clinical outcomes associated with EVT and to explore prognostic factors in patients with minor ischemic stroke caused by major intracranial arterial disease.

**Methods:**

This retrospective observational cohort study included consecutive adults with admission National Institutes of Health Stroke Scale (NIHSS) score ≤ 5 and severe intracranial stenosis or occlusion treated between January 2023 and December 2025. Patients were classified according to final treatment received as medical treatment or EVT, including direct and rescue EVT. The primary outcome was the full distribution of the 90-day modified Rankin Scale (mRS). Inverse probability of treatment weighting (IPTW), multivariable adjustment, and doubly robust analyses were used to address measured baseline imbalance.

**Results:**

Among 218 included patients, 128 received medical treatment and 90 underwent EVT, including 56 direct EVT and 34 rescue EVT cases. The median 90-day mRS score was 2 in the medical group and 1 in the EVT group. EVT was associated with a favorable ordinal mRS shift in the IPTW-weighted model (common odds ratio [OR], 1.69; 95% confidence interval [CI], 1.02–2.80; *P* = 0.043), with consistent findings in multivariable and doubly robust models. EVT was also associated with higher odds of 90-day mRS 0–2 (IPTW OR, 1.72; 95% CI, 1.04–2.85; *P* = 0.035) and 48-h NIHSS 0–1 (IPTW OR, 2.21; 95% CI, 1.18–4.14; *P* = 0.013). Symptomatic intracranial hemorrhage occurred in 3.1% and 5.6% of patients, respectively (OR, 1.82; 95% CI, 0.48–6.99; *P* = 0.493). Better collateral status and initial ischemic penumbra were associated with favorable outcome.

**Conclusions:**

In patients with minor ischemic stroke caused by severe intracranial stenosis or occlusion, EVT was associated with more favorable early neurological and 90-day functional outcomes after adjustment for measured confounding. Collateral status and ischemic penumbra may provide prognostic information, but their role in imaging-informed treatment selection requires prospective validation.

## 1 Introduction

Minor acute ischemic stroke is commonly defined by a low National Institutes of Health Stroke Scale (NIHSS) score, yet low symptom burden does not necessarily indicate low biological risk (Gkantzios et al., 2023). A substantial proportion of patients with mild deficits harbor major intracranial arterial occlusion or severe stenosis, and these lesions may still lead to infarct progression, early neurological deterioration (END), and unfavorable functional recovery (Gouta et al., 2025; Font et al., 2025). Recent multicenter observational studies and meta-analyses have shown that the prognosis of low-NIHSS large-vessel occlusion is heterogeneous and that the comparative value of endovascular treatment (EVT) vs. medical management remains uncertain, particularly in patients with proximal lesions or clinically eloquent occlusions (Safouris et al., 2023; Qin et al., 2023; Palazzo et al., 2023). One major reason for this uncertainty is that neurological severity at presentation may underestimate the degree of hemodynamic compromise. In patients with severe intracranial stenosis or occlusion, initial symptom preservation may reflect temporary compensation by collateral circulation rather than durable tissue stability (Zeng et al., 2025; Wang et al., 2024). Clinical studies published in recent years have suggested that cortical symptoms, lesion location, and low-NIHSS phenotypes alone are insufficient for treatment selection, while END remains a clinically important intermediary event linking early instability to poor long-term outcome. Recent registry analyses further suggest that selected patients with mild symptoms may still derive benefit from thrombectomy, especially when imaging or clinical features indicate a high-risk profile (Qiu et al., 2025a; Schwarz et al., 2023; Sabben et al., 2023).

This problem is particularly relevant in the setting of symptomatic intracranial atherosclerotic disease. Severe intracranial atherosclerotic stenosis is associated with recurrent ischemic events despite aggressive medical therapy, and the optimal role of lesion-directed endovascular treatment remains under active re-evaluation (Bin Aziz et al., 2025). The 2024 BASIS randomized clinical trial reported that balloon angioplasty plus aggressive medical management reduced the composite risk of stroke, death, or target-vessel revascularization compared with aggressive medical therapy alone in patients with symptomatic intracranial atherosclerotic stenosis. Subsequent studies have further examined sole balloon angioplasty, drug-coated balloons, drug-eluting stents, and other endovascular strategies, indicating that procedural feasibility is improving but that patient selection remains critical (Sun et al., 2024; Cao et al., 2025; Lin et al., 2025).

Accordingly, imaging-based risk stratification has become central to decision-making in this population (Avery et al., 2024). Computed tomography angiography can characterize responsible-vessel anatomy and collateral status, whereas perfusion imaging may identify ischemic penumbra and mismatch patterns that are not apparent from the admission NIHSS score alone (Kauw et al., 2020). Recent work on END prediction in symptomatic intracranial atherosclerotic stenosis has also reinforced the importance of integrating clinical, vascular, and hemodynamic variables rather than relying on symptom severity in isolation (Yang et al., 2025; Bu et al., 2025). Against this background, further investigation is warranted to clarify the prognostic and therapeutic implications of EVT in patients with minor ischemic stroke caused by severe stenosis or occlusion of a major intracranial artery.

## 2 Methods

### 2.1 Study design

This single-center retrospective observational cohort study screened consecutive patients treated at the institutional stroke center between January 1, 2023, and December 31, 2025. The study population comprised adults with minor acute ischemic stroke attributable to severe stenosis or occlusion of a major intracranial artery. Patients were eligible if they were aged 18 years or older; presented within 72 h after documented symptom onset; had acute ischemic stroke confirmed by clinical examination and computed tomography (CT) or magnetic resonance imaging; had severe stenosis or occlusion of the responsible intracranial internal carotid artery, middle cerebral artery M1 segment, basilar artery, or intracranial vertebral artery confirmed by CT angiography (CTA), magnetic resonance angiography, or digital subtraction angiography; and had an admission NIHSS score of 5 or less. Severe stenosis was defined as luminal narrowing of at least 70%. Patients were excluded if they had intracranial hemorrhage, substantial midline shift, chronic occlusion or arterial dissection of the responsible vessel, a pre-stroke modified Rankin Scale (mRS) score of 2 or greater, pregnancy or lactation, severe renal insufficiency, allergy to iodinated contrast material, a major coagulation disorder or high bleeding risk, a severe life-limiting comorbidity, anatomical or clinical unsuitability for endovascular treatment, or anticipated inability to complete treatment or follow-up. Because this retrospective cohort included all consecutive eligible patients treated during the predefined study period, the sample size was determined by the number of available cases, and no *a priori* sample-size calculation was performed. Effect estimates are presented with 95% confidence intervals to reflect statistical uncertainty. The number of parameters included in the multivariable models was restricted according to the available outcome events to reduce the risk of overfitting. This study was reviewed and approved by the ethics committee of our hospital. Informed consent was obtained from all participants or their legally authorized representatives. All procedures were conducted in accordance with relevant guidelines and regulations and the ethical principles of the Declaration of Helsinki. All data were anonymized before analysis and handled confidentially to protect participant privacy.

### 2.2 Clinical definitions and study time points

Minor ischemic stroke was operationally defined as an admission NIHSS score of ≤ 5, consistent with commonly used definitions of minor or mild ischemic stroke in clinical studies and guideline terminology referring to NIHSS 0–5 mild stroke symptoms. This threshold was predefined for this retrospective analysis to include patients with low but potentially disabling deficits, while acknowledging that stricter definitions, such as NIHSS ≤ 3 or ≤ 4, have also been used. The admission NIHSS score served as the common baseline neurological assessment for all between-group analyses. END was defined as an increase of ≥4 points in the NIHSS score within 7 days after stroke onset relative to the admission score.

The 48-h NIHSS score was defined as the assessment documented approximately 48 h after hospital admission. Neurological improvement was calculated as the admission NIHSS score minus the 48-h NIHSS score, with a positive value indicating improvement. Because a reduction of ≥4 points was not mathematically attainable for patients with admission NIHSS scores of 0–3, this endpoint was evaluated only among patients with admission NIHSS scores of 4–5 and was considered exploratory.

All clinical and procedural time points were retrospectively abstracted from the electronic medical records and institutional stroke and neurointerventional databases. The recorded workflow intervals included symptom onset to hospital admission, admission to initial CTA, symptom onset to initial CTA, admission to initial computed tomography perfusion (CTP), symptom onset to initial CTP, admission to intervention start, initial CTP to intervention start, symptom onset to intervention start, and procedure start to successful recanalization. For patients undergoing rescue EVT, the intervals from symptom onset to END, END to repeat imaging, and END to intervention start were additionally recorded.

Intervention start was defined as the documented start time of the endovascular procedure. The time of successful recanalization was defined as the first angiographically documented time point at which the predefined procedural success definition was met.

### 2.3 Treatment allocation and treatment protocols

Treatment was not randomly assigned. The treatment strategy was retrospectively classified according to the actual treatment received and was determined in routine clinical practice after neurological, vascular, and perfusion-imaging assessment. Decisions incorporated lesion characteristics, neurological course, target ischemic penumbra, collateral status, technical feasibility, clinician recommendations, the anticipated benefit–risk profile, and the informed preference of the patient or legally authorized representative.

Patients who did not undergo endovascular treatment received standard medical management, including intravenous thrombolysis when eligible, antiplatelet or anticoagulant therapy as clinically indicated, statin therapy, management of vascular risk factors, and stroke rehabilitation.

The EVT group included direct and rescue EVT. Direct EVT was performed before neurological deterioration in patients with a technically treatable responsible-vessel lesion and target ischemic penumbra on initial CTP. Rescue EVT was performed after early neurological deterioration in patients initially managed medically when repeat imaging showed persistent target ischemic penumbra and a technically treatable lesion. For analyses of END, rescue-EVT patients were classified according to their initial treatment strategy because END preceded the intervention.

Endovascular procedures were performed by experienced neurointerventionists according to institutional practice. Anesthesia was selected according to clinical and procedural requirements. Severe stenotic lesions were treated with balloon angioplasty, stent implantation, or both, whereas occlusive lesions were treated with mechanical thrombectomy, with rescue angioplasty or stenting when substantial residual stenosis, flow-limiting recoil, or re-occlusion was present. Periprocedural antithrombotic therapy was individualized according to the procedure, bleeding risk, and angiographic findings.

Apart from the endovascular procedure and associated perioperative management, EVT-treated patients received the same general medical and rehabilitative care as medically treated patients.

### 2.4 Data collection and imaging assessment

Baseline demographic, clinical, laboratory, imaging, and procedural data were retrospectively extracted from the electronic medical records and institutional stroke databases. Baseline laboratory variables were defined as the first available measurements obtained within 24 h after admission.

Initial imaging variables included lesion type, responsible-vessel location, collateral status, ischemic core volume, hypoperfusion volume, mismatch volume, mismatch ratio, and target ischemic penumbra. Only findings from the initial imaging examination were used in baseline comparisons. Repeat imaging obtained after early neurological deterioration in patients undergoing rescue EVT was evaluated separately as part of the rescue-treatment pathway.

CT perfusion data were processed using RAPID automated perfusion software (iSchemaView, Menlo Park, CA, USA). Ischemic core was defined as tissue with relative cerebral blood flow < 30% of that in contralateral normal tissue, and hypoperfused tissue was defined as tissue with *T*_max_ >6 s. Mismatch volume was calculated as hypoperfusion volume minus ischemic core volume, and mismatch ratio as hypoperfusion volume divided by ischemic core volume. Target ischemic penumbra was defined as an ischemic core volume ≤ 70 mL, a mismatch ratio ≥1.8, and a mismatch volume ≥15 mL. The same RAPID thresholds were applied to initial and repeat examinations.

Collateral circulation was retrospectively assessed on CTA source images and maximum-intensity-projection reconstructions using an institutional three-level qualitative scale. Collateral filling was classified as worse than, similar to, or better than that of the corresponding unaffected territory according to the extent and density of arterial opacification. The same assessment framework was applied to anterior- and posterior-circulation lesions. This institutional scale was not derived from a previously validated collateral-scoring system.

Imaging studies were retrospectively and independently reviewed by two experienced neuroradiologists who were blinded to the 90-day outcomes. Disagreements were resolved by consensus with a senior neuroradiologist. Individual pre-adjudication reader ratings were not retained in the retrospective database; therefore, inter-rater agreement statistics could not be calculated.

For EVT-treated patients, procedural data included anesthesia strategy, periprocedural antithrombotic treatment, endovascular technique, procedure duration, time to recanalization, procedural success, and procedure-related complications. Procedural success was defined as residual stenosis < 30% for severe stenotic lesions and a final modified Thrombolysis in Cerebral Infarction grade of 2b or 3 for occlusive lesions.

### 2.5 Outcome measures

The primary outcome was the full distribution of the mRS score at 90 days, assessed by trained investigators during outpatient follow-up or structured telephone interview.

Secondary outcomes included mRS 0–1 and mRS 0–2 at 90 days, NIHSS score at 48 h, change in NIHSS from admission to 48 h, NIHSS 0–1 at 48 h, and an NIHSS reduction of ≥4 points among patients with an admission NIHSS score of 4–5.

Safety outcomes included symptomatic intracranial hemorrhage and any intracranial hemorrhage within 48 h, recurrent ischemic stroke within 90 days, and all-cause mortality within 90 days. Symptomatic intracranial hemorrhage was defined as intracranial hemorrhage accompanied by an increase of ≥4 NIHSS points.

Among EVT-treated patients, additional outcomes included re-occlusion of the responsible vessel within 7 days and procedure-related complications.

The primary treatment comparison was based on final treatment received and classified patients as receiving medical treatment or any EVT. Because rescue EVT was performed after neurological deterioration, analyses of early neurological deterioration were based on the initial treatment strategy and compared direct EVT with initial medical management. The initial medical-management group included patients who remained medically treated and those who subsequently underwent rescue EVT.

### 2.6 Statistical analysis

Statistical analyses were performed using IBM SPSS Statistics, version 30.0 (IBM Corp., Armonk, NY, USA). Continuous variables are presented as mean ± standard deviation or median (interquartile range) and were compared using the independent-samples *t*-test or Mann–Whitney *U*-test, as appropriate. Categorical variables are presented as number (percentage) and were compared using Pearson's χ^2^-test or Fisher's exact test. Baseline balance was evaluated using absolute standardized mean differences (SMDs), with values < 0.10 indicating adequate balance. The primary analysis of the full 90-day mRS distribution used an inverse probability of treatment weighting (IPTW) proportional-odds ordinal logistic regression model. Propensity scores were estimated using pretreatment demographic, clinical, workflow, and imaging variables. Stabilized weights and robust standard errors were applied, and post-weighting balance was assessed using absolute SMDs. The proportional-odds assumption was formally tested. Unadjusted and conventional multivariable-adjusted proportional-odds models were reported as complementary analyses, and a doubly robust model combining IPTW with outcome regression was fitted to assess robustness. Binary outcomes were analyzed using logistic regression and reported as odds ratios with 95% confidence intervals. Weighted median differences were estimated for NIHSS outcomes. Safety outcomes were additionally summarized using absolute risk differences with Newcombe 95% confidence intervals. Exploratory multivariable logistic regression models were used to evaluate factors associated with favorable outcome and early neurological deterioration, with covariates selected according to clinical relevance and available outcome events. A separate IPTW sensitivity analysis compared direct EVT with medical treatment. Stratified analyses were conducted by lesion type, vascular territory, ischemic penumbra, and collateral status, with heterogeneity assessed using treatment-by-subgroup interaction terms. All tests were two-sided, and *P* < 0.05 was considered statistically significant.

## 3 Results

### 3.1 Patient screening and cohort construction

**Figure 1:**
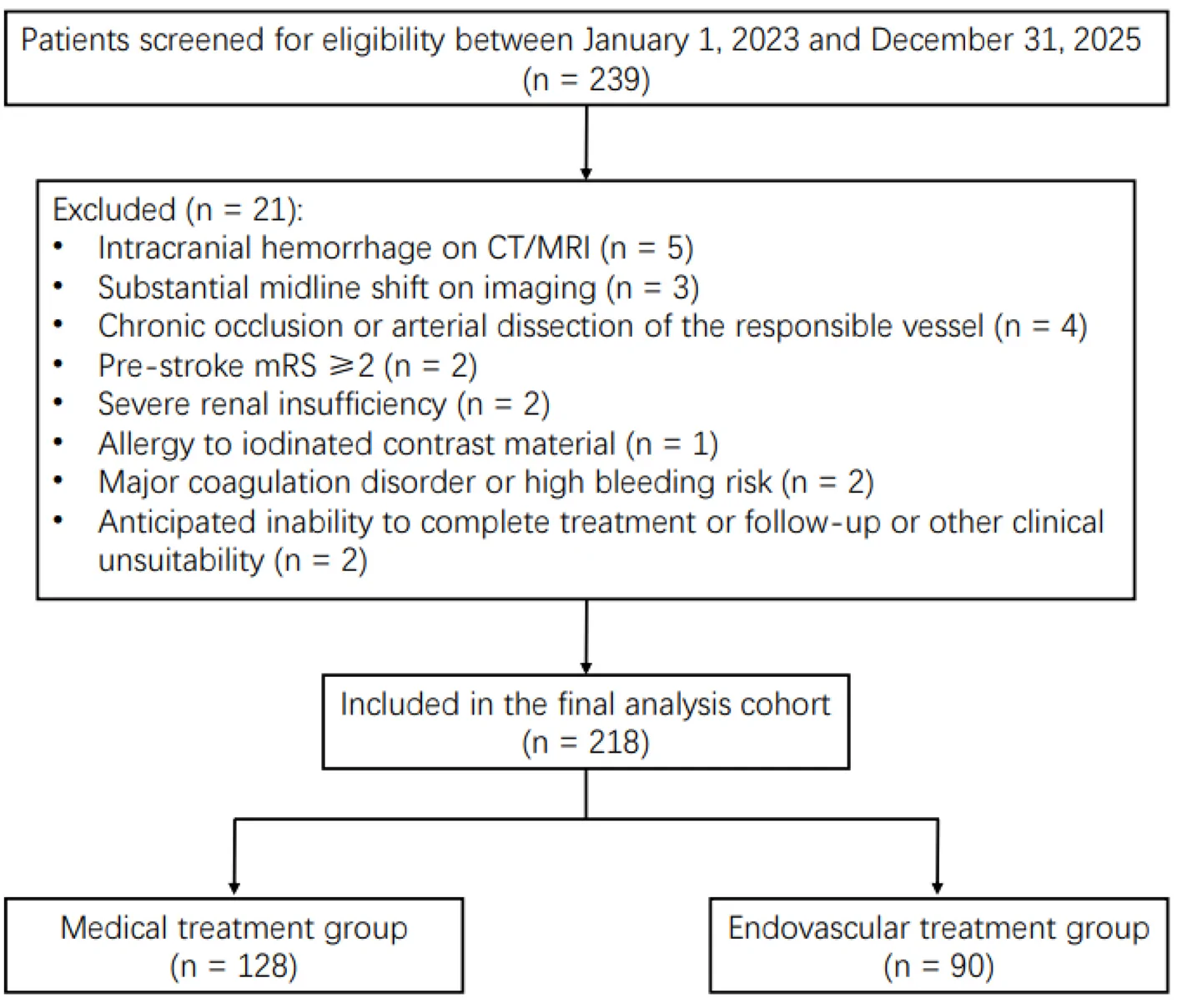
Patient screening and cohort construction.

Between January 1, 2023, and December 31, 2025, 239 consecutive patients with suspected minor acute ischemic stroke associated with severe stenosis or occlusion of a major intracranial artery were screened. Twenty-one patients were excluded according to the predefined eligibility criteria, leaving 218 patients in the final analysis cohort (Figure 1). A total of 218 patients were included. According to the final treatment received, 128 patients remained in the medical treatment group and 90 underwent EVT, including 56 who received direct EVT and 34 who received rescue EVT after early neurological deterioration. Accordingly, analyses based on the initial treatment strategy included 162 patients initially managed medically and 56 patients undergoing direct EVT. Complete baseline data and 90-day follow-up were available for all participants, with no loss to follow-up.

### 3.2 Baseline demographic and clinical characteristics

The study included 128 patients in the medical treatment group and 90 in the EVT group. Most demographic characteristics, vascular risk factors, pre-stroke medications, and admission blood pressure measures were similar between groups, with absolute SMDs generally below 0.10. However, imbalances were observed in neurological severity. The median admission NIHSS score was 2 (interquartile range [IQR], 2–3) in the medical treatment group and 3 (IQR, 2–4) in the EVT group. Admission NIHSS scores of 4–5 were present in 30 patients (23.4%) in the medical treatment group and 36 (40.0%) in the EVT group (absolute SMD, 0.362; *P* = 0.009). Conversely, NIHSS scores of 0–3 were recorded in 98 patients (76.6%) and 54 patients (60.0%), respectively. The complete admission NIHSS distribution showed that only patients with baseline scores of 4–5 had sufficient score range to achieve a reduction of at least 4 points (Table 1 and Supplementary Table S1).

**Table 1:** Baseline demographic and clinical characteristics before treatment.

Variable	Medical treatment group (*n* = 128)	EVT group (*n* = 90)	Absolute SMD	*P* value
Age, years	66.2 ± 10.8	65.1 ± 11.2	0.100	0.470
Male sex, *n* (%)	79 (61.7)	53 (58.9)	0.058	0.674
Smoking history, *n* (%)	49 (38.3)	37 (41.1)	0.058	0.674
Alcohol consumption history, *n* (%)	35 (27.3)	27 (30.0)	0.059	0.669
Hypertension, *n* (%)	83 (64.8)	60 (66.7)	0.038	0.780
Diabetes mellitus, *n* (%)	34 (26.6)	24 (26.7)	0.002	0.986
Previous ischemic stroke or TIA, *n* (%)	27 (21.1)	18 (20.0)	0.027	0.844
Atrial fibrillation, *n* (%)	12 (9.4)	11 (12.2)	0.092	0.500
Coronary artery disease, *n* (%)	21 (16.4)	17 (18.9)	0.065	0.634
Pre-stroke antiplatelet therapy, *n* (%)	31 (24.2)	22 (24.4)	0.005	0.969
Pre-stroke antihypertensive therapy, *n* (%)	54 (42.2)	40 (44.4)	0.046	0.740
Pre-stroke statin therapy, *n* (%)	25 (19.5)	20 (22.2)	0.066	0.629
Systolic blood pressure on admission, mmHg	151.6 ± 18.7	153.9 ± 20.4	0.118	0.398
Diastolic blood pressure on admission, mmHg	86.3 ± 11.1	87.5 ± 12.0	0.104	0.454
NIHSS score at symptom onset	2.1 ± 1.1	2.3 ± 1.2	0.174	0.212
Admission NIHSS, mean ± SD	2.50 ± 1.30	2.81 ± 1.40	0.231	0.093
Admission NIHSS, median (IQR)	2 (2–3)	3 (2–4)	—	0.093
Admission NIHSS 0–3, *n* (%)	98 (76.6)	54 (60.0)	0.362	0.009
Admission NIHSS 4–5, *n* (%)	30 (23.4)	36 (40.0)	0.362	0.009

Absolute SMD values ≥0.10 were considered indicative of potentially meaningful baseline imbalance. Only patients with an admission NIHSS score of 4–5 had sufficient baseline score range to achieve an improvement of ≥4 NIHSS points.

EVT, endovascular treatment; IQR, interquartile range; NIHSS, National Institutes of Health Stroke Scale; SD, standard deviation; SMD, standardized mean difference; TIA, transient ischemic attack.

### 3.3 Baseline laboratory, workflow, and imaging characteristics

No statistically significant between-group differences were observed in the recorded baseline laboratory measures. Substantial differences were present in workflow metrics. The mean onset-to-admission time was 18.6 ± 11.7 h in the medical treatment group and 8.2 ± 5.0 h in the EVT group (absolute SMD, 1.156; *P* < 0.001). The corresponding onset-to-initial computed tomography perfusion times were 20.9 ± 11.9 and 9.9 ± 5.1 h, respectively (absolute SMD, 1.202; *P* < 0.001).

The proportions of severe stenosis and occlusion were similar between groups. In contrast, collateral and perfusion characteristics were imbalanced. Collateral circulation worse than the contralateral side was observed in 40.6% of medically treated patients and 20.0% of EVT-treated patients, whereas collateral circulation better than the contralateral side was observed in 23.4% and 42.2%, respectively. The EVT group also had a larger mean mismatch volume (41.9 ± 25.6 vs. 34.2 ± 22.7 mL), a higher mismatch ratio (4.0 ± 1.5 vs. 3.1 ± 1.2), and a higher prevalence of target ischemic penumbra on initial perfusion imaging (86.7% vs. 61.7%). The ischemic core volume was similar between groups. RAPID-derived perfusion thresholds and operational imaging definitions are provided in the Supplementary material. Individual pre-adjudication reader-level ratings were unavailable, and inter-rater κ statistics could therefore not be estimated (Table 2 and Supplementary Table S2).

**Table 2:** Baseline laboratory findings, workflow metrics, and initial imaging characteristics.

Variable	Medical treatment group (*n* = 128)	EVT group (*n* = 90)	Absolute SMD	*P* value
Laboratory findings				
White blood cell count, × 10^9^/L	7.62 ± 2.11	7.48 ± 2.05	0.067	0.624
Hemoglobin, g/L	138.7 ± 16.2	137.3 ± 15.8	0.087	0.525
Platelet count, × 10^9^/L	214.5 ± 58.4	221.8 ± 61.7	0.122	0.380
International normalized ratio	1.01 ± 0.11	1.00 ± 0.10	0.095	0.486
Serum creatinine, μmol/L	76.8 ± 18.9	79.2 ± 19.4	0.125	0.365
Fasting blood glucose, mmol/L	7.34 ± 2.26	7.61 ± 2.41	0.116	0.405
Low-density lipoprotein cholesterol, mmol/L	2.63 ± 0.81	2.58 ± 0.77	0.063	0.645
Baseline workflow metrics				
Onset-to-admission time, h	18.6 ± 11.7	8.2 ± 5.0	1.156	< 0.001
Admission-to-initial CTA time, h	1.9 ± 0.8	1.5 ± 0.7	0.532	< 0.001
Onset-to-initial CTA time, h	20.5 ± 11.8	9.7 ± 5.1	1.188	< 0.001
Admission-to-initial CTP time, h	2.3 ± 0.9	1.8 ± 0.8	0.587	< 0.001
Onset-to-initial CTP time, h	20.9 ± 11.9	9.9 ± 5.1	1.202	< 0.001
Onset-to-intravenous thrombolysis time, h	3.52 ± 0.76	3.38 ± 0.69	0.193	0.490
Initial imaging characteristics				
Lesion type, overall *P* value	—	—	—	0.889
Severe stenotic lesion, *n* (%)	78 (60.9)	54 (60.0)	0.019	—
Occlusive lesion, *n* (%)	50 (39.1)	36 (40.0)	0.019	—
Responsible-vessel location, overall *P* value	—	—	—	0.813
Intracranial internal carotid artery, *n* (%)	28 (21.9)	24 (26.7)	0.112	—
Middle cerebral artery M1 segment, *n* (%)	54 (42.2)	33 (36.7)	0.113	—
Basilar artery, *n* (%)	26 (20.3)	18 (20.0)	0.008	—
Intracranial vertebral artery, *n* (%)	20 (15.6)	15 (16.7)	0.028	—
Collateral grade, overall *P* value	—	—	—	0.001
Worse than the contralateral side, *n* (%)	52 (40.6)	18 (20.0)	0.460	—
Equal to the contralateral side, *n* (%)	46 (35.9)	34 (37.8)	0.038	—
Better than the contralateral side, *n* (%)	30 (23.4)	38 (42.2)	0.408	—
Ischemic core volume, mL	8.4 ± 7.3	7.9 ± 6.8	0.071	0.605
Hypoperfusion volume, *T*_max_ >6 s, mL	42.6 ± 24.8	49.8 ± 27.5	0.275	0.049
Mismatch volume, mL	34.2 ± 22.7	41.9 ± 25.6	0.318	0.020
Mismatch ratio	3.1 ± 1.2	4.0 ± 1.5	0.663	< 0.001
Target ischemic penumbra on initial CTP, *n* (%)	79 (61.7)	78 (86.7)	0.595	< 0.001

CTA, computed tomography angiography; CTP, computed tomography perfusion; EVT, endovascular treatment; SMD, standardized mean difference; T_max_, time to maximum of the residue function.

### 3.4 Procedural and timeline characteristics of direct and rescue EVT

Among the 90 EVT-treated patients, 56 underwent direct EVT and 34 underwent rescue EVT after END. The onset-to-admission and onset-to-initial perfusion imaging intervals were similar between these subgroups. The median admission-to-intervention interval was 2.9 h (IQR, 2.4–3.4) for direct EVT and 8.6 h (IQR, 7.5–10.5) for rescue EVT (*P* < 0.001). The median onset-to-intervention interval was 9.6 h (IQR, 8.2–13.1) and 18.1 h (IQR, 12.4–20.2), respectively (*P* < 0.001).

Among rescue-EVT patients, the median onset-to-END interval was 15.5 h, the median END-to-repeat-imaging interval was 0.7 h, and the median END-to-intervention interval was 1.9 h. Procedure duration, procedure start-to-recanalization time, anesthesia strategy, and most procedural approaches were similar between subgroups. Stent implantation alone was more frequent in the rescue-EVT group than in the direct-EVT group (35.3% vs. 16.1%; *P* = 0.037). Procedural success was achieved in 89.3% and 82.4% of patients, respectively (Table 3).

**Table 3:** Procedural and timeline characteristics of direct and rescue EVT.

Variable	Direct EVT (*n* = 56)	Rescue EVT (*n* = 34)	Statistical test	*P* value
Onset-to-admission time, h, median (IQR)	6.7 (5.2–9.3)	7.3 (3.8–11.4)	Mann–Whitney *U* = 938.0	0.911
Onset-to-initial CTP time, h, median (IQR)	8.3 (6.7–11.8)	9.1 (6.7–13.0)	Mann–Whitney *U* = 882.0	0.563
Admission-to-intervention start, h, median (IQR)	2.9 (2.4–3.4)	8.6 (7.5–10.5)	Mann–Whitney *U* = 1.0	< 0.001
Initial CTP-to-intervention start, h, median (IQR)	1.2 (0.9–1.6)	6.6 (5.9–8.5)	Mann–Whitney *U* = 0.0	< 0.001
Onset-to-intervention start, h, median (IQR)	9.6 (8.2–13.1)	18.1 (12.4–20.2)	Mann–Whitney *U* = 314.0	< 0.001
Onset-to-END time, h, median (IQR)	—	15.5 (10.9–18.5)	—	—
END-to-repeat imaging time, h, median (IQR)	—	0.7 (0.5–0.9)	—	—
END-to-intervention start, h, median (IQR)	—	1.9 (1.2–2.4)	—	—
Procedure start-to-recanalization, min, median (IQR)	44 (32–59), *n* = 50	45 (33–52), *n* = 28	Mann–Whitney *U* = 708.0	0.938
Procedure duration, min, median (IQR)	69 (53–85)	70 (55–85)	Mann–Whitney *U* = 952.0	1.000
Local anesthesia or conscious sedation, *n* (%)	44 (78.6)	22 (64.7)	Pearson χ^2^ = 2.09	0.149
General anesthesia, *n* (%)	12 (21.4)	12 (35.3)	Pearson χ^2^ = 2.09	0.149
Preprocedural dual-antiplatelet loading, *n* (%)	42 (75.0)	29 (85.3)	Pearson χ^2^ = 1.35	0.246
Intraprocedural heparin administration, *n* (%)	24 (42.9)	18 (52.9)	Pearson χ^2^ = 0.86	0.353
Periprocedural glycoprotein IIb/IIIa inhibitor, *n* (%)	10 (17.9)	11 (32.4)	Pearson χ^2^ = 2.49	0.115
Balloon angioplasty alone, *n* (%)	24 (42.9)	10 (29.4)	Pearson χ^2^ = 1.63	0.202
Stent implantation alone, *n* (%)	9 (16.1)	12 (35.3)	Pearson χ^2^ = 4.37	0.037
Mechanical thrombectomy alone, *n* (%)	10 (17.9)	3 (8.8)	Fisher's exact test	0.356
Combined procedure, *n* (%)	13 (23.2)	9 (26.5)	Pearson χ^2^ = 0.12	0.727
Procedural success, *n* (%)	50 (89.3)	28 (82.4)	Pearson χ^2^ = 0.88	0.348
Procedure-related complications, *n* (%)	3 (5.4)	4 (11.8)	Fisher's exact test	0.419

Calculated only among the 78 patients achieving procedural success.

CTP, computed tomography perfusion; END, early neurological deterioration; EVT, endovascular treatment; IQR, interquartile range.

### 3.5 Primary 90-day functional outcome

The median 90-day mRS score was 2 (IQR, 1–3) in the medical treatment group and 1 (IQR, 0–2) in the EVT group (*P* = 0.004). The largest category-specific absolute difference occurred at mRS 0, which was achieved by 24 of 128 medically treated patients (18.8%) and 28 of 90 EVT-treated patients (31.1%).

In the primary IPTW-weighted proportional-odds analysis, EVT was associated with a shift toward lower disability across the full mRS distribution (common odds ratio [OR], 1.69; 95% confidence interval [CI], 1.02–2.80; *P* = 0.043). Similar estimates were obtained in the conventional multivariable-adjusted model (common OR, 1.74; 95% CI, 1.03–2.94; *P* = 0.039) and the doubly robust model (common OR, 1.67; 95% CI, 1.01–2.77; *P* = 0.047). The unadjusted proportional-odds model, reported for reference, yielded a common OR of 2.06 (95% CI, 1.26–3.35; *P* = 0.004). The proportional-odds assumption was not rejected in any model (all *P* > 0.30; Table 4).

**Table 4:** Primary 90-day functional outcome.

(A) Observed 90-day mRS distribution			
Outcome	Medical treatment group (*n* = 128)	EVT group (*n* = 90)	*P* value
90-day mRS, median (IQR)	2 (1–3)	1 (0–2)	0.004
mRS 0, *n* (%)	24 (18.8)	28 (31.1)	0.035
mRS 1, *n* (%)	31 (24.2)	28 (31.1)	0.259
mRS 2, *n* (%)	27 (21.1)	15 (16.7)	0.414
mRS 3, *n* (%)	24 (18.8)	10 (11.1)	0.126
mRS 4, *n* (%)	12 (9.4)	5 (5.6)	0.300
mRS 5, *n* (%)	6 (4.7)	2 (2.2)	0.475
mRS 6, *n* (%)	4 (3.1)	2 (2.2)	1.000
**(B) Ordinal shift analyses**			
**Model**	**Common OR for lower disability (95% CI)**	***P*** **value**	**Proportional-odds assumption**, ***P*** **value**
Unadjusted proportional-odds model	2.06 (1.26–3.35)	0.004	0.318
Multivariable-adjusted proportional-odds model	1.74 (1.03–2.94)	0.039	0.405
IPTW-weighted proportional-odds model	1.69 (1.02–2.80)	0.043	0.462
Doubly robust proportional-odds model	1.67 (1.01–2.77)	0.047	0.437

OR > 1 indicates a shift toward lower disability.

CI, confidence interval; EVT, endovascular treatment; IPTW, inverse probability of treatment weighting; IQR, interquartile range; mRS, modified Rankin Scale; OR, odds ratio.

### 3.6 Secondary functional and early neurological outcomes

At 90 days, mRS 0–1 was recorded in 43.0% of patients in the medical treatment group and 62.2% in the EVT group. The IPTW-weighted OR was 1.68 (95% CI, 1.02–2.76; *P* = 0.041). The corresponding proportions achieving mRS 0–2 were 64.1% and 78.9%, respectively, with an IPTW-weighted OR of 1.72 (95% CI, 1.04–2.85; *P* = 0.035).

The median 48-h NIHSS score was 1 (IQR, 0–2) in the medical treatment group and 0 (IQR, 0–1) in the EVT group. The IPTW-weighted median difference was −0.7 points (95% CI, −1.1 to −0.3; *P* = 0.001). The median improvement from admission to 48 h was 1 point (IQR, 0–2) and 2 points (IQR, 1–4), respectively, with a weighted median difference of 0.8 points (95% CI, 0.3–1.3; *P* = 0.002). An NIHSS score of 0–1 at 48 h was observed in 66.4% and 87.8% of patients, respectively (IPTW-weighted OR, 2.21; 95% CI, 1.18–4.14; *P* = 0.013).

Among patients with an admission NIHSS score of 4–5, a reduction of at least 4 points occurred in 23 of 30 medically treated patients (76.7%) and 34 of 36 EVT-treated patients (94.4%). The IPTW-weighted estimate was imprecise and did not reach statistical significance (OR, 3.94; 95% CI, 0.75–20.69; *P* = 0.105; Table 5).

**Table 5:** Secondary functional and early neurological outcomes.

Outcome	Medical treatment	EVT	Crude comparison or effect (95% CI)	Crude *P* value	IPTW-weighted effect (95% CI)	IPTW *P* value
90-day mRS 0–1, *n*/*N* (%)	55/128 (43.0)	56/90 (62.2)	OR 2.19 (1.26–3.79)	0.005	OR 1.68 (1.02–2.76)	0.041
90-day mRS 0–2, *n*/*N* (%)	82/128 (64.1)	71/90 (78.9)	OR 2.10 (1.13–3.90)	0.018	OR 1.72 (1.04–2.85)	0.035
NIHSS at 48 h, median (IQR)	1 (0–2)	0 (0–1)	Mann–Whitney *U* = 7,554.5	< 0.001	Weighted median difference −0.7 (−1.1 to −0.3)	0.001
Improvement in NIHSS from admission to 48 h, median (IQR)	1 (0–2)	2 (1–4)	Mann–Whitney *U* = 4,054.0	< 0.001	Weighted median difference 0.8 (0.3–1.3)	0.002
NIHSS 0–1 at 48 h, *n*/*N* (%)	85/128 (66.4)	79/90 (87.8)	OR 3.63 (1.75–7.54)	< 0.001	OR 2.21 (1.18–4.14)	0.013
NIHSS improvement ≥4 points among admission NIHSS 4–5, *n*/*N* (%)	23/30 (76.7)	34/36 (94.4)	OR 5.17 (0.99–27.16)	0.068	OR 3.94 (0.75–20.69)	0.105

Improvement was calculated as admission NIHSS minus 48-h NIHSS; positive values indicate improvement.

CI, confidence interval; EVT, endovascular treatment; IPTW, inverse probability of treatment weighting; IQR, interquartile range; mRS, modified Rankin Scale; NIHSS, National Institutes of Health Stroke Scale; OR, odds ratio.

### 3.7 Safety outcomes

Symptomatic intracranial hemorrhage within 48 h occurred in 4 of 128 patients (3.1%) in the medical treatment group and 5 of 90 patients (5.6%) in the EVT group. The absolute risk difference was 2.4 percentage points (95% CI, −3.2 to 9.5), and the OR was 1.82 (95% CI, 0.48–6.99; *P* = 0.493).

Any intracranial hemorrhage occurred in 5.5% and 10.0% of patients, corresponding to a risk difference of 4.5 percentage points (95% CI, −2.6 to 12.9). Recurrent ischemic stroke within 90 days occurred in 9.4% and 5.6%, respectively, with a risk difference of −3.8 percentage points (95% CI, −10.9 to 4.0). All-cause mortality occurred in 4.7% and 3.3%, respectively, with a risk difference of −1.4 percentage points (95% CI, −7.0 to 5.2). Confidence intervals for all safety outcomes were wide (Table 6).

**Table 6:** Safety outcomes.

Outcome	Medical treatment, *n*/*N* (%)	EVT, *n*/*N* (%)	Risk difference, percentage points (95% CI)	OR (95% CI)	*P* value
Symptomatic intracranial hemorrhage within 48 h	4/128 (3.1)	5/90 (5.6)	2.4 (−3.2 to 9.5)	1.82 (0.48–6.99)	0.493
Any intracranial hemorrhage within 48 h	7/128 (5.5)	9/90 (10.0)	4.5 (−2.6 to 12.9)	1.92 (0.69–5.36)	0.207
Recurrent ischemic stroke within 90 days	12/128 (9.4)	5/90 (5.6)	−3.8 (−10.9 to 4.0)	0.57 (0.19–1.67)	0.300
All-cause mortality within 90 days	6/128 (4.7)	3/90 (3.3)	−1.4 (−7.0 to 5.2)	0.70 (0.17–2.88)	0.739

Risk differences were calculated as EVT minus medical treatment.

CI, confidence interval; EVT, endovascular treatment; OR, odds ratio.

### 3.8 Treatment association and exploratory prognostic models

For the binary outcome of 90-day mRS 0–2, the crude final-treatment OR for EVT was 2.10 (95% CI, 1.13–3.90; *P* = 0.018). The corresponding estimates were 1.94 (95% CI, 1.01–3.73; *P* = 0.045) in the conventional multivariable model, 1.72 (95% CI, 1.04–2.85; *P* = 0.035) in the IPTW-weighted model, and 1.69 (95% CI, 1.02–2.81; *P* = 0.042) in the doubly robust model.

In the exploratory prognostic model, younger age, lower admission NIHSS score, collateral circulation better than the contralateral side, and the presence of initial ischemic penumbra were associated with higher odds of mRS 0–2 at 90 days. In the END model, direct EVT vs. initial medical management was not statistically associated with END (adjusted OR, 0.76; 95% CI, 0.39–1.49; *P* = 0.425). Higher admission NIHSS and occlusive lesions were associated with higher odds of END, whereas collateral circulation better than the contralateral side was associated with lower odds of END (Table 7).

**Table 7:** Treatment association and exploratory prognostic models.

(A) Association between EVT and favorable 90-day outcome				
Model	EVT effect estimate for mRS 0–2, OR (95% CI)		*P* value	
Crude final-treatment model	2.10 (1.13–3.90)		0.018	
Conventional multivariable final-treatment model	1.94 (1.01–3.73)		0.045	
IPTW-weighted final-treatment model	1.72 (1.04–2.85)		0.035	
Doubly robust final-treatment model	1.69 (1.02–2.81)		0.042	
**(B) Exploratory prognostic factors**				
**Variable**	**Favorable 90-day outcome, adjusted OR (95% CI)**	***P*** **value**	**END, adjusted OR (95% CI)**	***P*** **value**
Treatment exposure	Any EVT vs. final medical treatment: 1.94 (1.01–3.73)	0.045	Direct EVT vs. initial medical management: 0.76 (0.39–1.49)	0.425
Age, per 1-year increase	0.96 (0.93–0.99)	0.008	1.02 (0.98–1.05)	0.344
Admission NIHSS, per 1-point increase	0.67 (0.53–0.86)	0.001	1.55 (1.21–2.00)	0.001
Occlusion vs. severe stenosis	0.79 (0.41–1.52)	0.481	2.09 (1.08–4.05)	0.029
Collateral grade: equal vs. worse	1.41 (0.73–2.74)	0.306	0.69 (0.35–1.37)	0.291
Collateral grade: better vs. worse	2.28 (1.16–4.49)	0.017	0.37 (0.17–0.79)	0.010
Initial ischemic penumbra present	2.11 (1.02–4.36)	0.044	1.82 (0.98–3.39)	0.058
Atrial fibrillation	0.61 (0.22–1.71)	0.351	1.27 (0.47–3.45)	0.638
Onset-to-admission time, per hour	1.01 (0.98–1.03)	0.451	0.98 (0.95–1.01)	0.216

Candidate covariates were prespecified according to clinical relevance and availability before treatment. Each multivariable model included nine estimated parameters; 65 unfavorable 90-day outcomes and 84 END events yielded approximately 7.2 and 9.3 events per parameter, respectively.

CI, confidence interval; END, early neurological deterioration; EVT, endovascular treatment; IPTW, inverse probability of treatment weighting; mRS, modified Rankin Scale; NIHSS, National Institutes of Health Stroke Scale; OR, odds ratio.

### 3.9 Propensity-score balance and direct-EVT sensitivity analysis

Before weighting, substantial imbalances were present in admission NIHSS, workflow times, collateral status, mismatch ratio, and initial target penumbra. After IPTW, all reported absolute SMDs were below 0.06, with the largest residual SMD being 0.056 for onset-to-admission time.

In the sensitivity analysis restricted to direct EVT vs. medical treatment, the IPTW-weighted common OR for the 90-day ordinal mRS shift was 1.96 (95% CI, 1.08–3.56; *P* = 0.028). The weighted ORs were 2.02 (95% CI, 1.05–3.91; *P* = 0.035) for mRS 0–1, 2.44 (95% CI, 1.08–5.52; *P* = 0.032) for mRS 0–2, and 3.22 (95% CI, 1.23–8.43; *P* = 0.017) for NIHSS 0–1 at 48 h. Weighted safety models were not fitted because of the small numbers of adverse events (Supplementary Tables S3, S4).

### 3.10 Stratified analyses and direct-vs.-rescue EVT comparison

In analyses stratified by lesion type, the IPTW ORs for 90-day mRS 0–2 were 1.83 (95% CI, 0.80–4.19) for severe stenosis and 1.92 (95% CI, 0.75–4.94) for occlusion. No treatment-by-lesion-type interaction was identified (*P* for interaction = 0.930). Similarly, the association between direct EVT and END did not differ statistically between severe stenosis and occlusion (*P* for interaction = 0.584). In the vascular-territory analysis, the IPTW ORs for mRS 0–2 were 1.89 (95% CI, 0.91–3.93) for anterior-circulation disease and 1.78 (95% CI, 0.68–4.69) for posterior-circulation disease, with no statistical interaction (*P* for interaction = 0.907).

When direct and rescue EVT were compared, mRS 0–2 occurred in 85.7% and 67.6% of patients, respectively (OR, 2.87; 95% CI, 1.02–8.10; *P* = 0.042). The proportions achieving mRS 0–1 were 66.1% and 55.9%, respectively. Symptomatic intracranial hemorrhage, any intracranial hemorrhage, re-occlusion, procedure-related complications, recurrent ischemic stroke, and mortality were numerically more frequent in the rescue-EVT group, although the corresponding estimates had wide confidence intervals and none of these comparisons reached statistical significance (Supplementary Tables S5, S6, Supplementary Figure S1).

## 4 Discussion

This retrospective observational cohort study provides clinically relevant evidence on EVT in a challenging population of patients with minor ischemic stroke caused by large-vessel disease. Although these patients present with low NIHSS scores, severe intracranial stenosis or occlusion may expose them to early neurological deterioration and unfavorable functional recovery. The novelty of this study lies in its focus on imaging-selected minor stroke, the distinction between direct and rescue EVT pathways, and the integration of perfusion imaging, collateral status, lesion type, and propensity-score weighting into the outcome assessment. Rather than interpreting EVT as uniformly effective, our findings suggest that EVT was associated with more favorable functional and early neurological outcomes in selected patients with technically treatable lesions and evidence of salvageable tissue. These results may have practical value for multidisciplinary stroke decision-making, particularly when low initial neurological severity may underestimate the risk posed by large-vessel pathology. The study also highlights the importance of individualized assessment based on neurological course, vascular anatomy, perfusion mismatch, collateral circulation, procedural feasibility, and patient-level risk when considering EVT in minor ischemic stroke.

The primary functional analysis showed that EVT was associated with a favorable shift across the full 90-day mRS distribution after adjustment for baseline imbalance using IPTW. Importantly, the unadjusted estimate was attenuated after weighting and doubly robust adjustment, suggesting that part of the crude between-group difference was related to imaging-based treatment selection. Nevertheless, the direction of association remained consistent across the IPTW-weighted proportional-odds model, conventional multivariable model, and doubly robust model. This consistency supports the robustness of the observed association, while still requiring interpretation within the constraints of a nonrandomized design. The largest category-specific difference occurred at mRS 0, indicating that the observed ordinal shift was driven partly by a greater proportion of patients achieving complete functional recovery rather than by uniform separation across all disability levels. This pattern is clinically meaningful in minor stroke, where many patients already have relatively low baseline disability and the most relevant treatment distinction may be between complete recovery and mild residual symptoms. Secondary functional endpoints showed similar directions of association, including higher proportions of mRS 0–1 and mRS 0–2 at 90 days in the EVT group after weighting. These findings suggest that, among selected patients with minor stroke and large-vessel disease, EVT may be associated not only with avoidance of moderate disability but also with a higher likelihood of near-complete or complete functional independence (Chen et al., 2025b).

Early neurological outcomes further support the clinical relevance of the observed functional associations. The EVT group had lower 48-h NIHSS scores, greater early NIHSS improvement, and a higher proportion of NIHSS 0–1 after IPTW adjustment. These early neurological differences may represent a plausible pathway linking vascular intervention to later functional status, particularly in patients with persistent hypoperfusion or salvageable tissue. However, the analysis of NIHSS improvement of at least 4 points required careful interpretation because this threshold was mathematically unattainable for patients with admission NIHSS scores of 0–3. Restricting this exploratory endpoint to patients with admission NIHSS scores of 4–5 provided a more appropriate assessment of substantial early neurological improvement, although the estimate remained imprecise. The distinction between direct and rescue EVT also provides important clinical context. Patients undergoing rescue EVT had already experienced early neurological deterioration before intervention and therefore represented a biologically and clinically different subgroup from those treated directly. The direct-EVT sensitivity analysis showed associations in the same direction as the overall analysis, whereas the direct-vs.-rescue comparison suggested less favorable outcomes and numerically more adverse events among rescue-EVT patients. These findings emphasize that timing and clinical trajectory are central to interpreting EVT outcomes in minor stroke with large-vessel disease (PoŽar et al., 2025; Wang et al., 2025). They also support the need for close monitoring of initially medically managed patients with high-risk vascular or perfusion features, because neurological deterioration may identify a subgroup with poorer prognosis despite subsequent rescue intervention (Seners et al., 2021).

Our findings should be interpreted in the context of recent evidence regarding EVT for mild stroke with large-vessel occlusion. Recent meta-analyses reported no consistent overall functional advantage of EVT over medical management in this population and raised concerns regarding hemorrhagic risk, although some subgroup or non-crossover analyses suggested a possible association with excellent functional outcome (Xu et al., 2024; Chen et al., 2024). These findings underscore the uncertainty surrounding routine EVT for all patients with mild large-vessel occlusion and support a more selective, imaging-informed treatment strategy. In contrast to prior studies that largely considered large-vessel occlusion as a single entity, the present study included patients with severe intracranial stenosis or occlusion, incorporated perfusion-defined target penumbra and collateral status, and distinguished direct EVT from rescue EVT after early neurological deterioration. The multivariable findings were also clinically plausible: younger age, lower admission NIHSS score, better collateral circulation, and the presence of ischemic penumbra were associated with favorable 90-day outcome, whereas better collateral status was inversely associated with END. These observations are compatible with the concept that collateral flow may preserve threatened tissue and that penumbral imaging may help identify patients with residual tissue at risk. Recent studies in mild large- or medium-vessel occlusion populations similarly suggest that END is an important marker of poor outcome, with age, core infarct, occlusion site, isolated internal carotid artery occlusion, delayed antiplatelet therapy, and early neurological worsening reported as relevant prognostic factors (Qiu et al., 2025b; Hu et al., 2025; Kazmi et al., 2024). Our results extend these observations by suggesting that collateral grade and penumbral status may help refine risk stratification among initially mild patients. The comparison between direct and rescue EVT further indicates that these pathways should not be interpreted as equivalent clinical scenarios. Patients undergoing rescue EVT had already developed END and therefore represented a later and higher-risk ischemic trajectory (Chen et al., 2025a; Zhao et al., 2020). Consistent with reports on rescue EVT and meta-analytic concerns regarding crossover designs, our data do not argue against rescue intervention; rather, they suggest that rescue EVT may remain clinically valuable but may not fully offset the unfavorable context associated with neurological deterioration before reperfusion.

Several limitations should be acknowledged. First, this was a single-center retrospective observational study, and treatment allocation was influenced by clinical status, vascular and perfusion imaging, technical feasibility, clinician recommendations, and patient preference. Although IPTW and doubly robust adjustment were used to reduce measured imbalance, residual confounding and selection bias cannot be excluded; therefore, the findings should be interpreted as associations rather than definitive evidence of treatment efficacy. Second, safety events were infrequent, particularly symptomatic intracranial hemorrhage. No statistically significant differences were detected in hemorrhagic events, recurrent stroke, or mortality, but the wide confidence intervals indicate that clinically meaningful safety risks cannot be ruled out. Third, the cohort included both severe stenotic and occlusive lesions, introducing mechanistic heterogeneity. Although stratified analyses by lesion type, vascular territory, and treatment pathway were performed, these analyses were exploratory and limited by small subgroup sample sizes; thus, the absence of significant interaction should not be interpreted as evidence of equivalent associations across subgroups. Fourth, collateral status was assessed using an institutional qualitative scale rather than a validated collateral-scoring system, and inter-rater agreement could not be calculated, which may have introduced imaging misclassification. Finally, follow-up was limited to 90 days, preventing assessment of longer-term recovery, restenosis, recurrent ischemic events, and delayed complications. Despite these limitations, the study provides real-world, imaging-informed evidence that may help generate hypotheses for patient selection. Future multicenter prospective studies or randomized trials with standardized imaging protocols, validated collateral assessment, predefined criteria for direct and rescue EVT, larger subgroup samples, and longer follow-up are needed to clarify optimal treatment selection and the balance between functional outcomes and procedural risks.

## 5 Conclusion

In this retrospective observational cohort, endovascular treatment was associated with more favorable early neurological and 90-day functional outcomes in selected patients with minor ischemic stroke caused by severe intracranial stenosis or occlusion. No statistically significant difference in major adverse events was detected, although safety estimates were limited by sparse events. Collateral status, ischemic penumbra, and early neurological deterioration may help inform imaging-based risk stratification, but their role in treatment selection requires prospective validation. Prospective multicenter studies and randomized trials are needed to confirm these findings and define the optimal candidate population.

## Data Availability

The raw data supporting the conclusions of this article will be made available by the authors, without undue reservation.
