# Supplementary material for: Endovascular treatment and clinical outcomes in minor ischemic stroke due to large-vessel disease: a retrospective study
Source: Front Neurosci. 2026 Aug 21;20:1885422. doi: 10.3389/fnins.2026.1885422 (PMC13541921; doi:10.3389/fnins.2026.1885422)
Supplement: Supplementary file 1 [file Data_Sheet_1.docx]

Supplementary Table S1. Admission NIHSS distribution and floor-effect assessment

| Admission NIHSS score | Medical treatment, n (%) | EVT, n (%) |
| --- | --- | --- |
| 0 | 6 (4.7) | 3 (3.3) |
| 1 | 23 (18.0) | 17 (18.9) |
| 2 | 40 (31.3) | 19 (21.1) |
| 3 | 29 (22.7) | 15 (16.7) |
| 4 | 20 (15.6) | 27 (30.0) |
| 5 | 10 (7.8) | 9 (10.0) |
| NIHSS 0–3 | 98 (76.6) | 54 (60.0) |
| NIHSS 4–5 | 30 (23.4) | 36 (40.0) |
| Total | 128 (100.0) | 90 (100.0) |

Abbreviations: EVT, endovascular treatment; NIHSS, National Institutes of Health Stroke Scale.

Supplementary Table S2. RAPID-derived imaging definitions and imaging reliability

Panel A. Operational definitions

| Variable | Operational definition |
| --- | --- |
| Ischemic core | Relative cerebral blood flow <30% of contralateral normal tissue |
| Hypoperfused tissue | Tmax >6 seconds |
| Mismatch volume | Hypoperfusion volume minus ischemic core volume |
| Mismatch ratio | Hypoperfusion volume divided by ischemic core volume |
| Target ischemic penumbra | Core ≤70 mL, mismatch ratio ≥1.8, and mismatch volume ≥15 mL |
| Repeat CTP | Processed using the same RAPID version and thresholds as initial CTP |
| Severe stenosis | Luminal narrowing ≥70% |
| Procedural success for stenosis | Residual stenosis <30% |
| Procedural success for occlusion | Final mTICI grade 2b or 3 |

Panel B. Availability of inter-rater reliability data

| Imaging variable | Independent reader-level records available | Agreement statistic |
| --- | --- | --- |
| Collateral grade | No | Not estimable |
| Lesion type | No | Not estimable |
| Responsible-vessel location | No | Not estimable |
| Target penumbra status | No | Not estimable |
| Procedural success | No | Not estimable |

Abbreviations: CTP, computed tomography perfusion; mTICI, modified Thrombolysis in Cerebral Infarction; RAPID, Rapid Processing of Perfusion and Diffusion; Tmax, time to maximum of the residue function.

Supplementary Table S3. Covariate balance before and after IPTW

| Variable | Medical unweighted | EVT unweighted | SMD before | Medical weighted | EVT weighted | SMD after |
| --- | --- | --- | --- | --- | --- | --- |
|  |  |  |  |  |  |  |
| Age, years | 66.2 ± 10.8 | 65.1 ± 11.2 | 0.100 | 65.8 ± 10.9 | 65.7 ± 10.8 | 0.009 |
| Male sex, % | 61.7 | 58.9 | 0.058 | 60.6 | 60.2 | 0.008 |
| Smoking history, % | 38.3 | 41.1 | 0.058 | 39.4 | 39.8 | 0.008 |
| Alcohol consumption, % | 27.3 | 30 | 0.059 | 28.5 | 28.8 | 0.007 |
| Hypertension, % | 64.8 | 66.7 | 0.038 | 65.6 | 65.8 | 0.004 |
| Diabetes mellitus, % | 26.6 | 26.7 | 0.002 | 26.6 | 26.5 | 0.002 |
| Previous stroke or TIA, % | 21.1 | 20 | 0.027 | 20.7 | 20.6 | 0.003 |
| Atrial fibrillation, % | 9.4 | 12.2 | 0.092 | 10.6 | 10.9 | 0.010 |
| Coronary artery disease, % | 16.4 | 18.9 | 0.065 | 17.4 | 17.6 | 0.005 |
| Admission NIHSS | 2.50 ± 1.30 | 2.81 ± 1.40 | 0.231 | 2.65 ± 1.34 | 2.68 ± 1.37 | 0.022 |
| Systolic blood pressure, mmHg | 151.6 ± 18.7 | 153.9 ± 20.4 | 0.118 | 152.5 ± 19.1 | 152.8 ± 19.6 | 0.015 |
| Onset-to-admission time, h | 18.6 ± 11.7 | 8.2 ± 5.0 | 1.156 | 13.7 ± 9.1 | 13.2 ± 8.8 | 0.056 |
| Admission-to-initial CTA time, h | 1.9 ± 0.8 | 1.5 ± 0.7 | 0.532 | 1.72 ± 0.76 | 1.69 ± 0.75 | 0.040 |
| Onset-to-initial CTP time, h | 20.9 ± 11.9 | 9.9 ± 5.1 | 1.202 | 15.6 ± 9.4 | 15.1 ± 9.1 | 0.054 |
| Intravenous thrombolysis, % | 21.9 | 26.7 | 0.112 | 24 | 24.5 | 0.012 |
| Occlusive lesion, % | 39.1 | 40 | 0.019 | 39.6 | 39.8 | 0.004 |
| Anterior circulation, % | 64.1 | 63.3 | 0.016 | 63.8 | 63.6 | 0.004 |
| Posterior circulation, % | 35.9 | 36.7 | 0.016 | 36.2 | 36.4 | 0.004 |
| Collateral grade worse, % | 40.6 | 20 | 0.460 | 30.6 | 30.2 | 0.009 |
| Collateral grade equal, % | 35.9 | 37.8 | 0.038 | 36.8 | 36.6 | 0.004 |
| Collateral grade better, % | 23.4 | 42.2 | 0.408 | 32.6 | 33.2 | 0.013 |
| Ischemic core volume, mL | 8.4 ± 7.3 | 7.9 ± 6.8 | 0.071 | 8.15 ± 7.1 | 8.10 ± 7.0 | 0.007 |
| Hypoperfusion volume, mL | 42.6 ± 24.8 | 49.8 ± 27.5 | 0.275 | 45.7 ± 25.3 | 46.2 ± 25.9 | 0.019 |
| Mismatch ratio | 3.1 ± 1.2 | 4.0 ± 1.5 | 0.663 | 3.53 ± 1.31 | 3.57 ± 1.35 | 0.030 |
| Initial target penumbra, % | 61.7 | 86.7 | 0.595 | 74.3 | 74.8 | 0.012 |

Abbreviations: CTA, computed tomography angiography; CTP, computed tomography perfusion; EVT, endovascular treatment; IPTW, inverse probability of treatment weighting; NIHSS, National Institutes of Health Stroke Scale; SMD, standardized mean difference; TIA, transient ischemic attack.

Supplementary Table S4. Direct EVT versus medical treatment sensitivity analysis

Panel A. Clinical outcomes

| Outcome | Medical treatment (n=128) | Direct EVT (n=56) | Crude effect (95% CI) | Crude P value | Exploratory direct-EVT-specific IPTW effect (95% CI) | IPTW P value |
| --- | --- | --- | --- | --- | --- | --- |
| 90-day ordinal mRS shift | — | — | Common OR 2.44 (1.39–4.28) | 0.002 | Common OR 1.96 (1.08–3.56) | 0.028 |
| 90-day mRS 0–1, n/N (%) | 55/128 (43.0) | 37/56 (66.1) | OR 2.58 (1.34–4.97) | 0.004 | OR 2.02 (1.05–3.91) | 0.035 |
| 90-day mRS 0–2, n/N (%) | 82/128 (64.1) | 48/56 (85.7) | OR 3.37 (1.47–7.73) | 0.003 | OR 2.44 (1.08–5.52) | 0.032 |
| NIHSS 0–1 at 48 h, n/N (%) | 85/128 (66.4) | 52/56 (92.9) | OR 6.58 (2.23–19.39) | <0.001 | OR 3.22 (1.23–8.43) | 0.017 |
| Symptomatic ICH within 48 h, n/N (%) | 4/128 (3.1) | 2/56 (3.6) | OR 1.15 (0.20–6.46) | 1.000 | Not modeled | — |
| Any ICH within 48 h, n/N (%) | 7/128 (5.5) | 4/56 (7.1) | OR 1.33 (0.37–4.74) | 0.738 | Not modeled | — |
| Recurrent ischemic stroke within 90 days, n/N (%) | 12/128 (9.4) | 2/56 (3.6) | OR 0.36 (0.08–1.66) | 0.233 | Not modeled | — |
| All-cause mortality within 90 days, n/N (%) | 6/128 (4.7) | 1/56 (1.8) | OR 0.37 (0.04–3.14) | 0.677 | Not modeled | — |

Panel B. Covariate balance for the direct-EVT-specific IPTW analysis

| Variable | Medical treatment, unweighted (n=128) | Direct EVT, unweighted (n=56) | Absolute SMD before weighting | Medical treatment, weighted | Direct EVT, weighted | Absolute SMD after weighting |
| --- | --- | --- | --- | --- | --- | --- |
| Age, years | 66.2 ± 10.8 | 64.8 ± 10.6 | 0.131 | 65.7 ± 10.9 | 65.5 ± 10.7 | 0.019 |
| Male sex, % | 61.7 | 60.7 | 0.021 | 60.500 | 60.8 | 0.006 |
| Smoking history, % | 38.3 | 41.1 | 0.057 | 39.500 | 39.9 | 0.008 |
| Alcohol consumption, % | 27.3 | 30.4 | 0.067 | 29.100 | 29.5 | 0.009 |
| Hypertension, % | 64.8 | 66.1 | 0.026 | 65.600 | 65.9 | 0.006 |
| Diabetes mellitus, % | 26.6 | 26.8 | 0.005 | 26.700 | 26.9 | 0.005 |
| Previous ischemic stroke or TIA, % | 21.1 | 19.6 | 0.036 | 20.500 | 20.9 | 0.010 |
| Atrial fibrillation, % | 9.4 | 10.7 | 0.045 | 10.100 | 10.3 | 0.007 |
| Coronary artery disease, % | 16.4 | 17.9 | 0.039 | 17.100 | 17.4 | 0.008 |
| Admission NIHSS, mean ± SD | 2.50 ± 1.30 | 2.93 ± 1.36 | 0.323 | 2.69 ± 1.34 | 2.72 ± 1.35 | 0.022 |
| Admission NIHSS 4–5, % | 23.4 | 46.4 | 0.497 | 43.800 | 44.1 | 0.006 |
| Systolic blood pressure, mmHg | 151.6 ± 18.7 | 154.7 ± 20.0 | 0.16 | 152.8 ± 19.2 | 153.2 ± 19.5 | 0.021 |
| Onset-to-admission time, h | 18.6 ± 11.7 | 7.8 ± 4.5 | 1.218 | 12.9 ± 8.8 | 12.4 ± 8.6 | 0.057 |
| Admission-to-initial CTA time, h | 1.9 ± 0.8 | 1.4 ± 0.7 | 0.665 | 1.71 ± 0.76 | 1.68 ± 0.74 | 0.040 |
| Onset-to-initial CTP time, h | 20.9 ± 11.9 | 9.5 ± 4.8 | 1.256 | 14.8 ± 9.3 | 14.3 ± 9.0 | 0.055 |
| Intravenous thrombolysis, % | 21.9 | 30.4 | 0.194 | 25.400 | 25.9 | 0.011 |
| Occlusive lesion, % | 39.1 | 39.3 | 0.005 | 39.500 | 39.8 | 0.006 |
| Anterior circulation, % | 64.1 | 64.3 | 0.005 | 63.900 | 64.2 | 0.006 |
| Posterior circulation, % | 35.9 | 35.7 | 0.005 | 36.100 | 35.8 | 0.006 |
| Collateral grade worse than contralateral side, % | 40.6 | 14.3 | 0.618 | 27.500 | 27.1 | 0.009 |
| Collateral grade equal to contralateral side, % | 35.9 | 35.7 | 0.005 | 36.800 | 37.1 | 0.006 |
| Collateral grade better than contralateral side, % | 23.4 | 50 | 0.573 | 35.700 | 35.8 | 0.002 |
| Ischemic core volume, mL | 8.4 ± 7.3 | 7.5 ± 6.6 | 0.129 | 7.95 ± 7.00 | 7.88 ± 6.90 | 0.010 |

Abbreviations: CI, confidence interval; CTA, computed tomography angiography; CTP, computed tomography perfusion; EVT, endovascular treatment; ICH, intracranial hemorrhage; IPTW, inverse probability of treatment weighting; mRS, modified Rankin Scale; NIHSS, National Institutes of Health Stroke Scale; OR, odds ratio; SD, standard deviation; SMD, standardized mean difference; TIA, transient ischemic attack.

Supplementary Table S5. Stratified analyses by lesion type and vascular territory

Panel A. Outcomes according to final treatment received, stratified by lesion type

| Outcome | Lesion type | Final medical treatment | Any EVT | Effect estimate (95% CI) | P value | P for interaction |
| --- | --- | --- | --- | --- | --- | --- |
| 90-day mRS 0–2 | Severe stenosis | 55/78 (70.5) | 45/54 (83.3) | IPTW OR 1.83 (0.80–4.19) | 0.154 | 0.930 |
|  | Occlusion | 27/50 (54.0) | 26/36 (72.2) | IPTW OR 1.92 (0.75–4.94) | 0.174 |  |
| Symptomatic ICH | Severe stenosis | 2/78 (2.6) | 2/54 (3.7) | Crude OR 1.46 (0.20–10.71) | 1.000 | 0.772 |
|  | Occlusion | 2/50 (4.0) | 3/36 (8.3) | Crude OR 2.18 (0.35–13.78) | 0.645 |  |
| Any ICH | Severe stenosis | 3/78 (3.8) | 4/54 (7.4) | Crude OR 2.00 (0.43–9.32) | 0.443 | 0.943 |
|  | Occlusion | 4/50 (8.0) | 5/36 (13.9) | Crude OR 1.85 (0.46–7.46) | 0.482 |  |

Panel B. END according to initial treatment strategy, stratified by lesion type

| Outcome | Lesion type | Initial medical management | Direct EVT | Adjusted OR for direct EVT (95% CI) | P value | P for interaction |
| --- | --- | --- | --- | --- | --- | --- |
| END | Severe stenosis | 35/98 (35.7) | 8/34 (23.5) | 0.61 (0.24–1.55) | 0.300 | 0.584 |
|  | Occlusion | 31/64 (48.4) | 10/22 (45.5) | 0.91 (0.34–2.45) | 0.852 |  |

Panel C. Functional outcome according to final treatment received, stratified by vascular territory

| Outcome | Vascular territory | Final medical treatment | Any EVT | IPTW OR (95% CI) | P value | P for interaction |
| --- | --- | --- | --- | --- | --- | --- |
| 90-day mRS 0–2 | Anterior circulation | 54/82 (65.9) | 46/57 (80.7) | 1.89 (0.91–3.93) | 0.088 | 0.907 |
|  | Posterior circulation | 28/46 (60.9) | 25/33 (75.8) | 1.78 (0.68–4.69) | 0.242 |  |

Abbreviations: CI, confidence interval; END, early neurological deterioration; EVT, endovascular treatment; ICH, intracranial hemorrhage; IPTW, inverse probability of treatment weighting; mRS, modified Rankin Scale; OR, odds ratio.

Supplementary Table S6. Direct versus rescue EVT outcomes and safety

| Outcome | Direct EVT (n=56) | Rescue EVT (n=34) | Effect estimate (95% CI) | P value |
| --- | --- | --- | --- | --- |
| 90-day mRS 0–1 | 37 (66.1) | 19 (55.9) | OR 1.54 (0.64–3.69) | 0.334 |
| 90-day mRS 0–2 | 48 (85.7) | 23 (67.6) | OR 2.87 (1.02–8.10) | 0.042 |
| NIHSS 0–1 at 48 h | 52 (92.9) | 27 (79.4) | OR 3.37 (0.91–12.54) | 0.094 |
| Procedural success | 50 (89.3) | 28 (82.4) | OR 1.79 (0.53–6.06) | 0.348 |
| Symptomatic ICH within 48 h | 2 (3.6) | 3 (8.8) | OR 0.38 (0.06–2.42) | 0.362 |
| Any ICH within 48 h | 4 (7.1) | 5 (14.7) | OR 0.45 (0.11–1.79) | 0.290 |
| Re-occlusion within 7 days | 2 (3.6) | 4 (11.8) | OR 0.28 (0.05–1.61) | 0.194 |
| Procedure-related complications | 3 (5.4) | 4 (11.8) | OR 0.42 (0.09–2.03) | 0.419 |
| Recurrent ischemic stroke within 90 days | 2 (3.6) | 3 (8.8) | OR 0.38 (0.06–2.42) | 0.362 |
| All-cause mortality within 90 days | 1 (1.8) | 2 (5.9) | OR 0.29 (0.03–3.34) | 0.554 |

Abbreviations: CI, confidence interval; EVT, endovascular treatment; ICH, intracranial hemorrhage; mRS, modified Rankin Scale; NIHSS, National Institutes of Health Stroke Scale; OR, odds ratio.

Supplementary Figure S1. Forest plot of the association between endovascular treatment and favorable functional outcome across the overall cohort, sensitivity analysis, and prespecified subgroups.

Favorable functional outcome was defined as a modified Rankin Scale score of 0–2 at 90 days. Odds ratios greater than 1 favor endovascular treatment. The overall estimate, lesion-type and vascular-territory subgroup estimates, and direct-EVT sensitivity estimate were derived from inverse probability of treatment weighting analyses. Estimates according to target ischemic penumbra and collateral circulation were unadjusted because of the limited subgroup sample sizes. Horizontal lines represent 95% confidence intervals, and the vertical dashed line indicates an odds ratio of 1. Interaction P values are shown for lesion type, vascular territory, target ischemic penumbra, and collateral circulation.

Abbreviations: CI, confidence interval; EVT, endovascular treatment; IPTW, inverse probability of treatment weighting; mRS, modified Rankin Scale; OR, odds ratio.
